# MERS-CoV ORF4b is a virulence factor involved in the inflammatory pathology induced in the lungs of mice

**DOI:** 10.1371/journal.ppat.1010834

**Published:** 2022-09-21

**Authors:** Melissa Bello-Perez, Jesús Hurtado-Tamayo, Ricardo Requena-Platek, Javier Canton, Pedro José Sánchez-Cordón, Raúl Fernandez-Delgado, Luis Enjuanes, Isabel Sola

**Affiliations:** 1 Department of Molecular and Cell Biology, National Center of Biotechnology (CNB-CSIC), Campus Universidad Autónoma de Madrid, Darwin, Madrid, Spain; 2 Veterinary Pathology Department, Animal Health Research Center (CISA), National Institute of Research, Agricultural and Food Technology (INIA-CSIC), Valdeolmos, Madrid, Spain; The University of Hong Kong, HONG KONG

## Abstract

No vaccines or specific antiviral drugs are authorized against Middle East respiratory syndrome coronavirus (MERS-CoV) despite its high mortality rate and prevalence in dromedary camels. Since 2012, MERS-CoV has been causing sporadic zoonotic infections in humans, which poses a risk of genetic evolution to become a pandemic virus. MERS-CoV genome encodes five accessory proteins, 3, 4a, 4b, 5 and 8b for which limited information is available in the context of infection. This work describes 4b as a virulence factor *in vivo*, since the deletion mutant of a mouse-adapted MERS-CoV-Δ4b (MERS-CoV-MA-Δ4b) was completely attenuated in a humanized DPP4 knock-in mouse model, resulting in no mortality. Attenuation in the absence of 4b was associated with a significant reduction in lung pathology and chemokine expression levels at 4 and 6 days post-infection, suggesting that 4b contributed to the induction of lung inflammatory pathology. The accumulation of 4b in the nucleus *in vivo* was not relevant to virulence, since deletion of its nuclear localization signal led to 100% mortality. Interestingly, the presence of 4b protein was found to regulate autophagy in the lungs of mice, leading to upregulation of BECN1, ATG3 and LC3A mRNA. Further analysis in MRC-5 cell line showed that, in the context of infection, MERS-CoV-MA 4b inhibited autophagy, as confirmed by the increase of p62 and the decrease of ULK1 protein levels, either by direct or indirect mechanisms. Together, these results correlated autophagy activation in the absence of 4b with downregulation of a pathogenic inflammatory response, thus contributing to attenuation of MERS-CoV-MA-Δ4b.

## Author summary

Middle East respiratory syndrome coronavirus (MERS-CoV) is a highly pathogenic human coronavirus that is still causing sporadic zoonotic infections in humans. Knowledge of virus-host interactions is required to develop specific protection strategies. This manuscript identifies 4b MERS-CoV protein as a virulence factor *in vivo*, independent of its nuclear localization signal, and demonstrates that the presence of 4b induces a proinflammatory response and inhibits autophagy. These results highlight the relevance of the activation of the inflammatory response and the inhibition of autophagy in the virulence of MERS-CoV, and suggest 4b as a candidate antiviral target.

## Introduction

Middle East respiratory syndrome coronavirus (MERS-CoV) emerged in Saudi Arabia in 2012. Since then, MERS-CoV has spread to 27 countries and 2591 cases have been reported at the end of July 2022 (http://www.emro.who.int/health-topics/mers-cov/mers-outbreaks.html). MERS-CoV has the highest mortality rate (>35%) among the seven known human coronaviruses, with clinical manifestations including acute respiratory distress syndrome, septic shock, multi-organ failure and death [1–3].

CoV virulence has been associated with specific genes that are non-essential for virus replication, but are involved in the regulation of virus-host interactions [4, 5]. These genes encode accessory proteins, which are variable in number, sequence and function in different coronaviruses. MERS-CoV encodes five accessory proteins 3, 4a, 4b, 5 and 8b [6–8], which have been functionally characterized mainly by overexpression, in the absence of infection. ORF4a [9], ORF4b [10], ORF5 and ORF8b [11] were described as IFNα/β antagonists [7], while ORF4a was reported as a potent inhibitor of the PKR-mediated stress response pathway [12]. Functional information is even more limited in MERS-CoV *in vivo* infections. Deletion of ORFs 3, 4a, 4b and 5 together in a mouse adapted MERS-CoV led to an attenuated phenotype [5, 13], although the individual contribution to attenuation of each ORF is still unknown. ORF5 is the only MERS-CoV accessory protein for which *in vivo* function has been described. Protein 5 has been shown to modulate the innate immune response, contributing to reduce lethality, since MERS-CoV-Δ5 was more virulent than the parental MERS-CoV in a transgenic mouse model of infection [14]. Additionally, the expression of 4b by the mouse coronavirus rJ2.2. did not significantly enhanced virulence in mice [11], suggesting it was not a virulence factor in the context of rJ2.2 infection.

The pandemic potential of CoVs, recently confirmed by SARS-CoV-2 [15], highlights the importance of studying the molecular bases of CoV pathogenesis, still largely unknown. This knowledge will inform the search of antiviral drugs and vaccine development, still not available for clinical use in MERS-CoV infections [16]. The identification of MERS-CoV individual accessory proteins involved in pathogenesis represents the first step to decipher its mechanism and will help to define novel host antiviral targets. Furthermore, deletion of virulence genes from the viral genome may contribute to the development of attenuated vaccine candidates [13].

Recently, our laboratory described that 4b protein interacts with karyopherin alpha 4 (KPNA4) in the context of infection in Huh-7 cells, leading to the competitive inhibition of KPNA4 binding to the p65 subunit of nuclear factor kb (NF-kB). This interaction prevents translocation of NF-kB to the nucleus and therefore reduces the expression of NF-kB dependent pro-inflammatory cytokines [17]. However, the relevance of 4b protein to MERS-CoV infection *in vivo* has not been described yet.

Autophagy is a natural process by which unnecessary or dysfunctional cytoplasmatic components (such as damaged organelles or denatured proteins) are engulfed by a double-membrane vesicle called autophagosome. Subsequently, the autophagosome fuses with a lysosome to form the autolysosome, whose content is degraded and recycled [18–21]. Basal levels of autophagy are required to maintain cellular homeostasis. However, due to the role of autophagy in the clearing of intracellular components, viruses have developed mechanisms to prevent autophagosome formation, which might lead to virus destruction [22]. Other respiratory viruses, like Influenza virus [23, 24] rhinoviruses, coxsackieviruses [25] and coronaviruses [26, 27], have been reported to modulate autophagy. Nevertheless, the relevance of the autophagy pathway in CoV infections is still controversial and further research is warranted [28–30].

This paper describes the contribution of ORF4b individually to MERS-CoV-MA pathogenesis and shows that 4b protein is a virulence factor that promotes the expression of pro-inflammatory cytokines and chemokines in mice lungs. The presence of 4b protein was also associated with autophagy inhibition both *in vivo* and in lung MRC5 cell lines, suggesting that autophagy deficiency was contributing to MERS-CoV-MA inflammatory pathology.

## Results

### Generation of MERS-CoV-MA-Δ4b and MERS-CoV-MA-mNLS mutants

**Fig 1:**
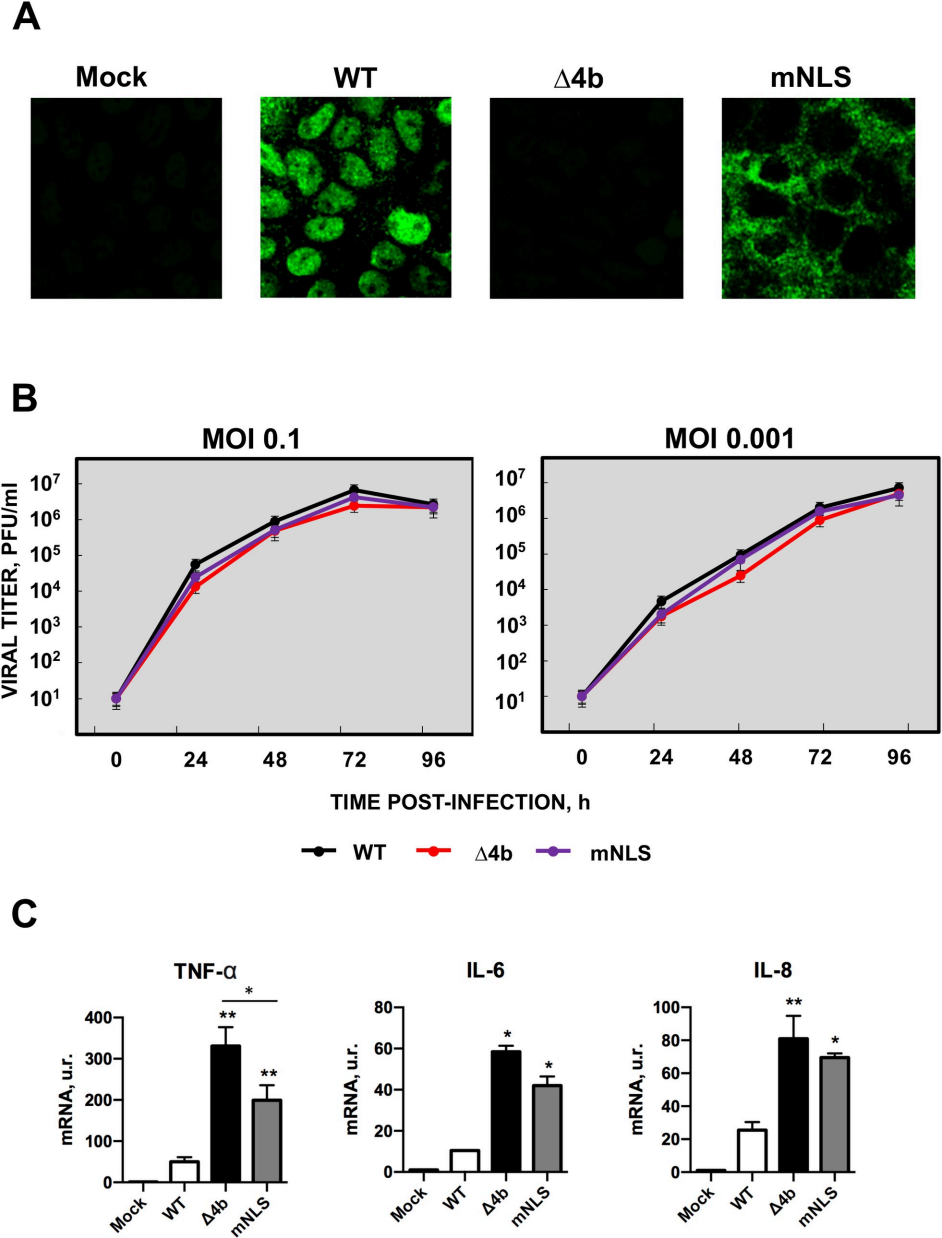
Characterization of MERS-CoV-MA-Δ4b and MERS-CoV-MA-mNLS mutants. (A) Huh-7 cells were infected with MERS-CoV-MA, MERS-CoV-MA-Δ4b or MERS-CoV-MA-mNLS at a m.o.i. of 0.1 for 24 h and the localization of 4b (in green) was analyzed by confocal microscopy. (B) Subconfluent monolayers of Huh-7 were infected with wild-type (black), Δ4b (red) or mNLS (purple) at a m.o.i. of 0.1 or 0.001. Culture supernatants were collected at 24, 48, 72 and 96 h p.i. and titrated by plaque assay. The average of two independent experiments is represented. (C) Pro-inflammatory response induced in Huh-7 by the infection with WT, Δ4b or mNLS. RNA was collected at 24 h p.i and quantified by RT-qPCR. Error bars represent standard deviations of the mean (n = 3). Differences with WT group were analyzed by Student’s t test: *, p-value < 0.05; **, p-value < 0.01.

The mouse-adapted mutants MERS-CoV-MA-Δ4b and MERS-CoV-MA-mNLS were engineered by replacing in pBAC-MERS-CoV-MA [14], 4b genomic sequences by chemically synthesized fragments that included 4b deletion or mutation to alanine of its nuclear localization signal (NLS), as previously described [17]. The absence of 4b protein and the cytoplasmic localization of 4b during infection of Huh-7 cells with MERS-CoV-MA-Δ4b or MERS-CoV-MA-mNLS, respectively, were confirmed by confocal microscopy **(Fig 1A)**. MERS-CoV-MA-Δ4b and MERS-CoV-MA-mNLS grew in Huh-7 cells to slightly lower titers than the parental MERS-CoV-*WT* at both low (0.001) and high (0.1) m.o.i., although these differences in growth kinetics were not statistically significant **(Fig 1B)**, indicating that the absence of 4b protein or mutation of 4b NLS did not significantly affect virus growth in cell cultures. The complete deletion of gene 4b or the mutation of the NLS in the MERS-CoV-MA induced in Huh7 cells a significant increase in mRNA levels of pro-inflammatory cytokines (TNF-α, IL-6 and IL-8) at 24 h p.i., as previously observed with the human versions of MERS-CoV-Δ4b and MERS-CoV- mNLS [17] **(Fig 1C)**.

### Influence of MERS-CoV-MA 4b protein and its NLS domain on *in vivo* virulence

**Fig 2:**
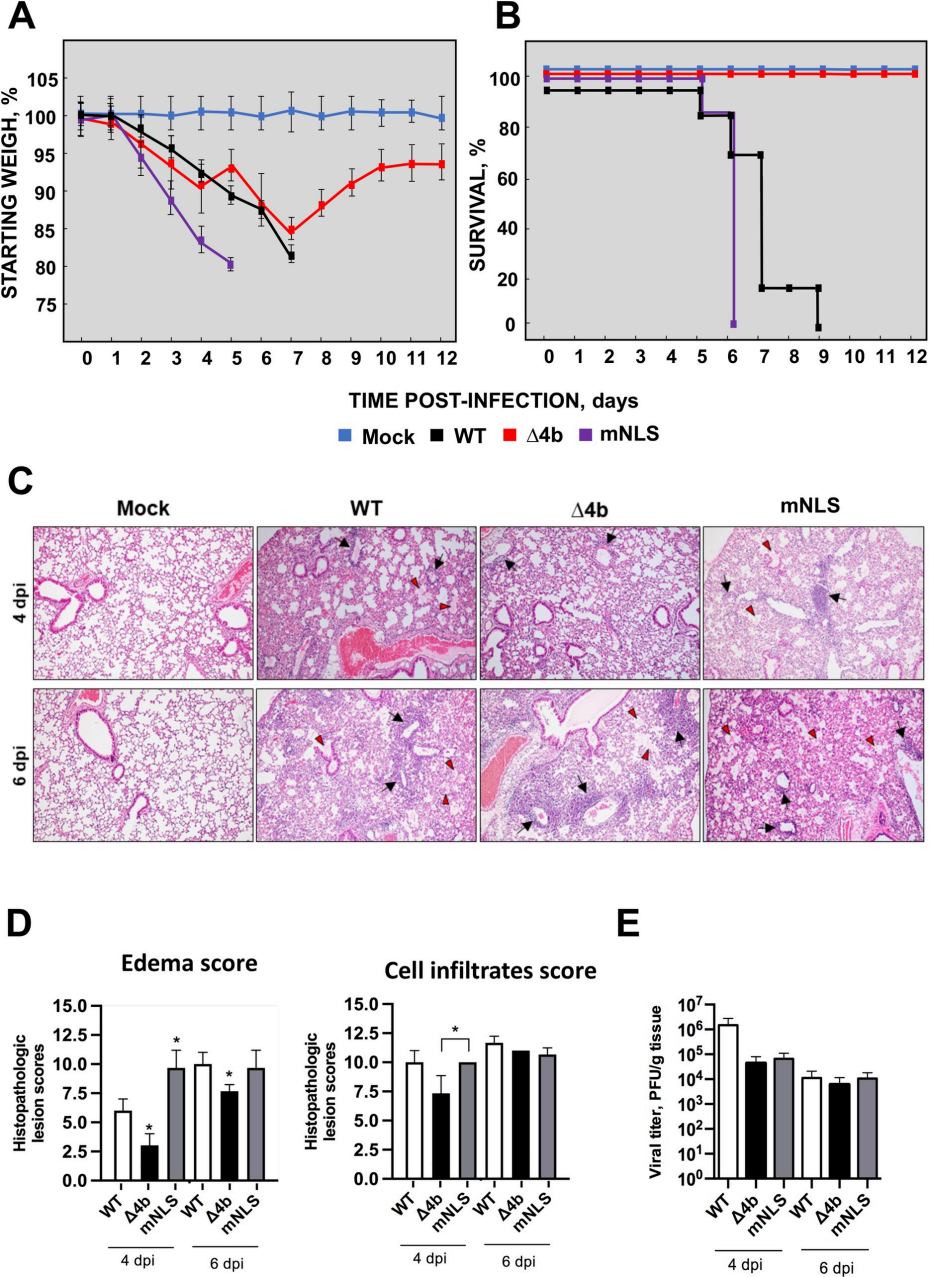
Virulence of MERS-CoV-MA-Δ4b and MERS-CoV-MA-mNLS in mice. 30-week-old hDPP4-KI mice were intranasally inoculated with 5x10^4^ pfu of MERS-CoV-MA-WT (WT), MERS-CoV-MA-Δ4b (Δ4b) or MERS-CoV-MA-mNLS (mNLS) viruses. Weight loss (A) and survival (B) were monitored for 12 days. Error bars represent standard deviations of the mean (n = 5 mice per group). (C) Representative images of pulmonary histopathological lesions (H&E staining; magnification 10x) observed in mice euthanized at 4 and 6 dpi. Alveolar septal thickening, perivascular and peribronchiolar cuffs populated by lymphocytes (black arrows) and alveolar edema (red arrowheads) were among the most characteristic inflammatory lesions observed. (D) Scores associated with edema and cell infiltrates at 4 and 6 dpi. (E) Viral titers in the lungs were determined by plaque assay at 4 and 6 dpi (n = 3).

To determine the effect of 4b protein in pathogenesis, hDPP4-KI mice were intranasally infected with a lethal dose (5x10^4^ PFU/animal) of MERS-CoV-MA-WT (n = 11), MERS-CoV-MA-Δ4b (n = 11), MERS-CoV-MA-mNLS (n = 11) or mock-infected (n = 11). In contrast to mock-infected mice, all infected animals lost weight from the second or third day post-infection **(Fig 2A)**. MERS-CoV-MA-WT infection led to a severe weight loss, associated with the death of all the animals by 9 dpi **(Fig 2B)**. In contrast, MERS-CoV-MA-Δ4b-infected mice recovered weight and all of them survived until the end of the experiment (12 dpi). The presence of the NLS in 4b protein was not required for virulence, since MERS-CoV-MA-mNLS infected mice lost weight as those infected with the WT virus **(Fig 2A)** and all of them died by day 6 post-infection **(Fig 2B).** Together, these results demonstrated that 4b protein was a virulence factor and the 4b NLS was not a main determinant of virulence.

**Fig 3:**
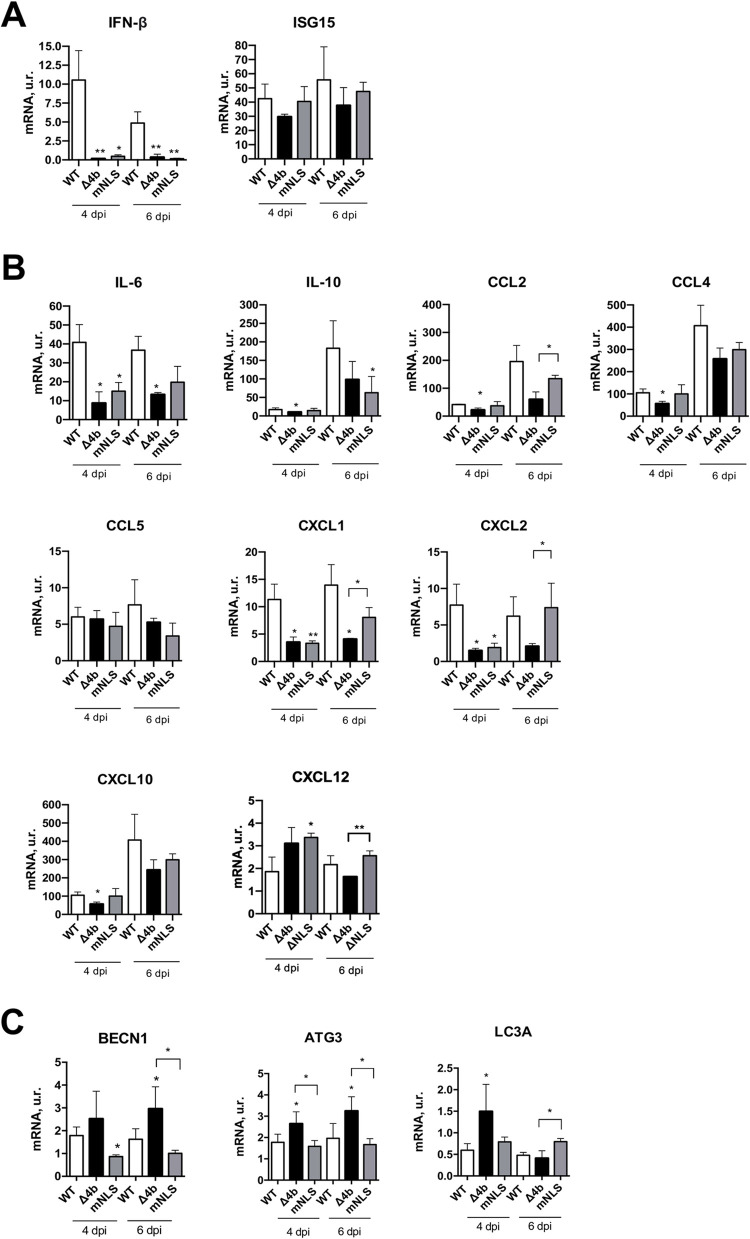
Innate immune response to MERS-CoV-MA-Δ4b in mice. hDPP4-KI mice were infected with 5x10^4^ PFU of MERS-CoV-MA-WT, MERS-CoV-MA-Δ4b or MERS-CoV-MA-mNLS virus. Mice were sacrificed at 4 and 6 dpi and the RNA of the lungs was extracted for gene expression assays. mRNA expression levels of genes related to the IFN response (A), the pro-inflammatory response (B), and autophagy (C) were quantified by RT-qPCR. Error bars represent standard deviations of the mean (n = 3). Differences between groups were analyzed by Student’s t test: *, p-value < 0.1; **, p-value < 0.01.

Pulmonary histopathology in MERS-CoV-infected mice was scored from lung sections (Fig 2C and 2D) at 4 and 6 dpi, according to the presence of edema, including perivascular and alveolar edema and alveolar haemorrhages; and cell infiltrates, including interstitial, peribronchiolar, perivascular and alveolar mononuclear infiltrates. Mice infected with MERS-CoV-MA-Δ4b showed significantly reduced edema as compared to mice infected with virulent MERS-CoV-MA-mNLS or MERS-CoV-WT, both at 4 and 6 dpi. This reduction in MERS-CoV-MA-Δ4b histopathology was mainly associated with milder and occasional lesions, in contrast to the severe and extensive perivascular and alveolar edema observed in mice infected MERS-CoV-MA-mNLS and MERS-CoV-WT (Fig 2C). Moreover, infection with MERS-CoV-MA-Δ4b led to significantly lower cell infiltration at 4 dpi than infection with MERS-CoV-MA-mNLS (Fig 2D). At 4 dpi, alveolar cell infiltrates were mainly macrophages, while peribronchiolar and perivascular infiltrates were almost entirely lymphocytes. At 6 dpi, the abundance of cell infiltrates increased and lymphocytes represented the majority of mononuclear cell infiltrates in the lungs. This increase was compatible with the significantly higher levels of lymphocyte and monocyte chemoattractants CCL2, CXCL10 and CXCL12 (Fig 3B) [31]. At 4 and 6 dpi all mice, including those infected with the attenuated MERS-CoV-MA-Δ4b, showed similar weight losses. However, only MERS-CoV-MA-Δ4b-infected mice recovered weight from 7 dpi onwards, leading to 100% survival. These results suggested that the milder lung pathology observed in MERS-CoV-MA-Δ4b-infected mice at early stages of infection, especially the alveolar edema and, to a lesser extent, inflammatory infiltrates, might be related to downregulation of pro-inflammatory cytokines. The lower levels of pro-inflammatory chemokines CCL2, CCL4, CXCL1, CXCL2, CXCL10 and CXCL12 (Fig 3B) in MERS-CoV-MA-Δ4b-infected lungs would have prevented severe and prolonged damage, thus promoting the recovery of normal lung physiology in later stages, as the virus was cleared and inflammatory mediators decreased. These milder lesions would also contribute to subsequent weight recovery and eventually, to the survival of mice. On the contrary, the severe prolonged lung damage observed from 4 dpi in mice infected with MERS-CoV-MA-WT or MERS-CoV-MA-mNLS, most likely induced by higher levels of pro-inflammatory cytokines, would cause an irreversible weight loss and the death of mice.

The viral titers of MERS-CoV-MA-Δ4b and MERS-CoV-MA-mNLS in the lungs of mice were similar at both 4 and 6 dpi. At 4 dpi, their growth *in vivo* was around ten-fold lower than that of MERS-CoV-WT, while at 6 dpi viral titers were similar for all of them **(Fig 2D)**. Altogether, these results indicated that attenuation of MERS-CoV-MA-Δ4b was not associated with lower viral titers, since it grew as efficiently as the virulent MERS-CoV-MA-mNLS, but to the absence of 4b protein, which is a virulence factor *in vivo*.

### Inflammatory response induced by MERS-CoV-MA-Δ4b *in vivo*

The extent of the inflammatory response and the timing of interferon (IFN) response have been associated with the severe pulmonary pathology caused by MERS-CoV [32]. To analyze the innate immune response induced in the lungs of mice by MERS-CoV-MA-Δ4b, a set of mRNAs encoding cytokines (IL-6, IL-10), chemokines (CCL2, CCL4, CCL5, CXCL1, CXCL2 and CXCL10) and interferon related genes (IFN-β and ISG-15) were quantified by qRT-PCR at 4 and 6 dpi. Infection with MERS-CoV-MA-Δ4b and MERS-CoV-mNLS led to reduced levels of IFN-β and IL-6 compared to MERS-CoV-MA-WT at 4 and 6 dpi, suggesting that the nuclear localization of 4b was necessary *in vivo* to induce the expression of IFN-β and IL-6. These results suggested that the expression of IFN-β and pro-inflammatory cytokine IL-6 at these time points was not a major determinant of MERS-CoV-MA virulence, since similar reduced IFN-β and IL-6 expression was observed for virulent MERS-CoV-MA-mNLS and attenuated MERS-CoV-MA-Δ4b.

Compared to virulent MERS-CoV-MA-WT and MERS-CoV-MA-mNLS infection, attenuated MERS-CoV-MA-Δ4b induced, either at 4 or 6 dpi, lower levels of CCL2, CCL4 and CXCL10, which are monocyte and macrophage chemoattractants, and CXCL1 and CXCL2, which are neutrophil chemoattractants **(Fig 3A and 3B)**. These results correlated with less inflammatory changes and cell infiltrates in the lungs of MERS-CoV-MA-Δ4b infected mice **(Fig 2)**, as compared to MERS-CoV-MA-WT and MERS-CoV-MA-mNLS, particularly at 4 dpi. Inflammatory changes and mononuclear cell infiltrates were previously associated with lethality in MERS-CoV infection *in vivo* [32]. Together, these results indicated that the presence of 4b with or without the NLS, contributed to the induction of high levels of inflammatory chemokines (CCL2, CXCL1 and CXCL-2), not observed in MERS-CoV-MA-Δ4b infected lungs. These chemokine expression levels preceded the accumulation of cell infiltrates in the lungs (**Fig 2B**).

### Contribution of MERS-CoV-MA 4b protein to autophagy in the lungs of mice

There is evidence that autophagy is involved in viral infections [33] and elevated autophagy can downregulate inflammation induced, either chemically [34] or by virus infection [35], in the lungs.

**Fig 4:**
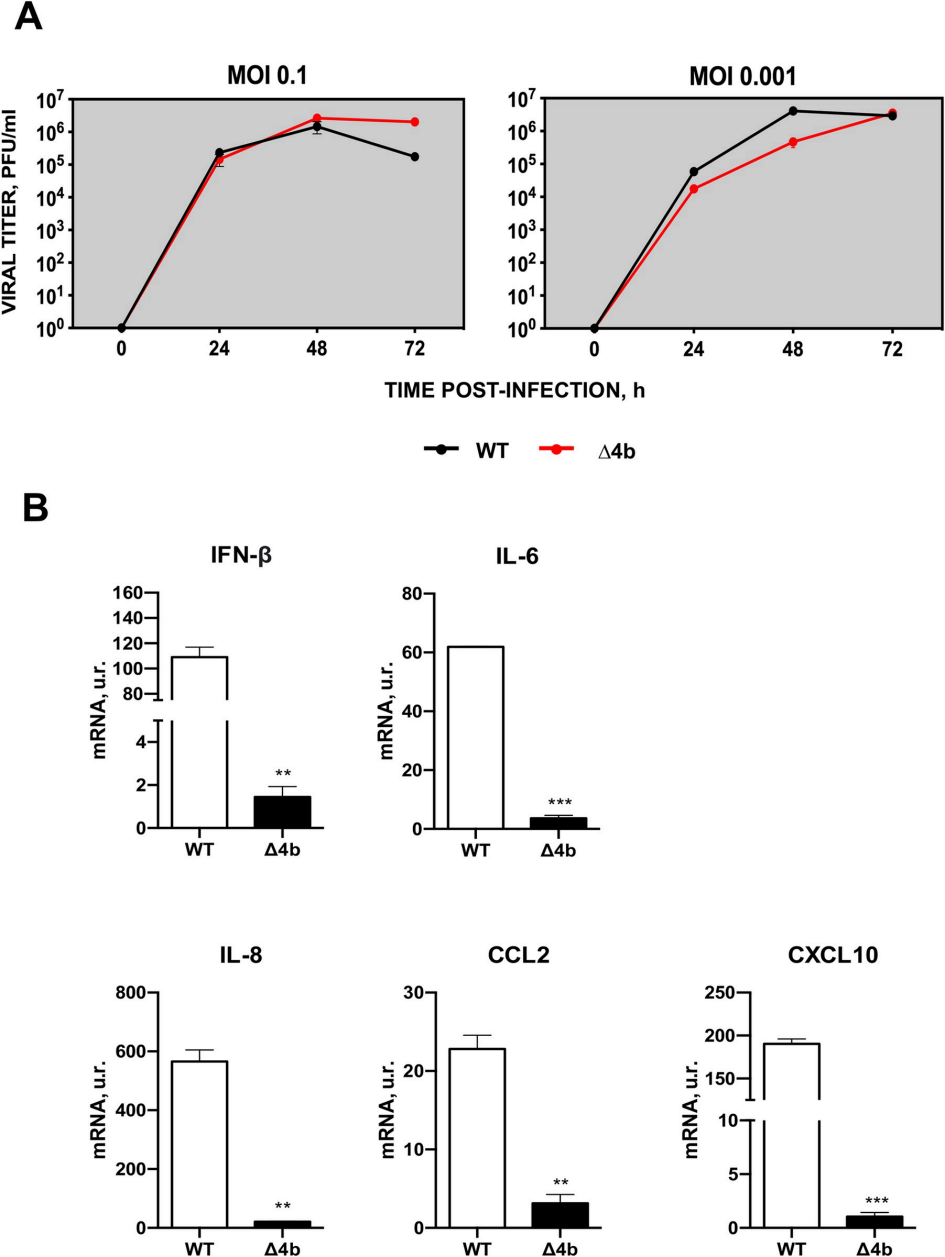
Characterization of MERS-CoV-MA-WT and MERS-CoV-MA-Δ4b in MRC-5 cells. (A) Subconfluent monolayers of MRC-5 were infected with wild-type or Δ4b at a m.o.i. of 0.1 or 0.001. Culture supernatants were collected at 24, 48 and 72 h p.i. and titrated by plaque assay. The average of two independent experiments is represented. (B) The mRNA expression levels of genes related to the IFN or the pro-inflammatory responses were quantified by RT-qPCR in MRC-5 cells either mock-infected or infected with WT or Δ4b viruses at m.o.i. 1 and 24 h p.i. Error bars represent standard deviations of the mean (n = 3). Differences were analyzed by Student’s t test: *, p-value < 0.05; **, p-value < 0.01.

It has been described that autophagy efficiently reduces the replication of MERS-CoV in cell cultures [29]. However, it has not been shown whether MERS-CoV-induced inflammatory pathology is associated with autophagy. In order to study this potential relationship, the expression levels of a set of autophagy-related genes, BECN1, ATG3 and LC3A, were analyzed by qPCR in the lungs of infected mice. Deletion of 4b in attenuated MERS-CoV-MA-Δ4b increased mRNA levels of autophagy mediators BECN1, ATG3 and LC3A, as compared to infection with virulent viruses MERS-CoV-MA-WT and MERS-CoV-MA-mNLS, suggesting that 4b protein was contributing to the modulation of autophagy *in vivo*, independently of the presence or the absence of 4b-NLS, **(Fig 3C)**. Therefore, the attenuation of MERS-CoV-MA-Δ4b was associated with a reduction in lung inflammation and higher expression of autophagy genes, suggesting that autophagy regulation (activation or inhibition) could contribute to MERS-CoV virulence. To further study this mechanism, human pulmonary MRC-5 cells were infected with MERS-CoV-MA or MERS-CoV-MA-Δ4b, since in this cell line both viruses grow similarly (Fig 4A) and the pro-inflammatory innate immune response observed in mice during MERS-CoV-MA infection, in the presence or the absence of 4b protein, is reproduced (Fig 4B). Deletion of 4b protein led to a significant reduction in the expression of IFNß, pro-inflammatory cytokines (IL-6 and IL-8) and chemokines (CCL2 and CXCL10) in relation to MERS-CoV-MA-WT infection.

### Contribution of MERS-CoV-MA 4b protein to autophagy in MRC-5 human lung cells

**Fig 5:**
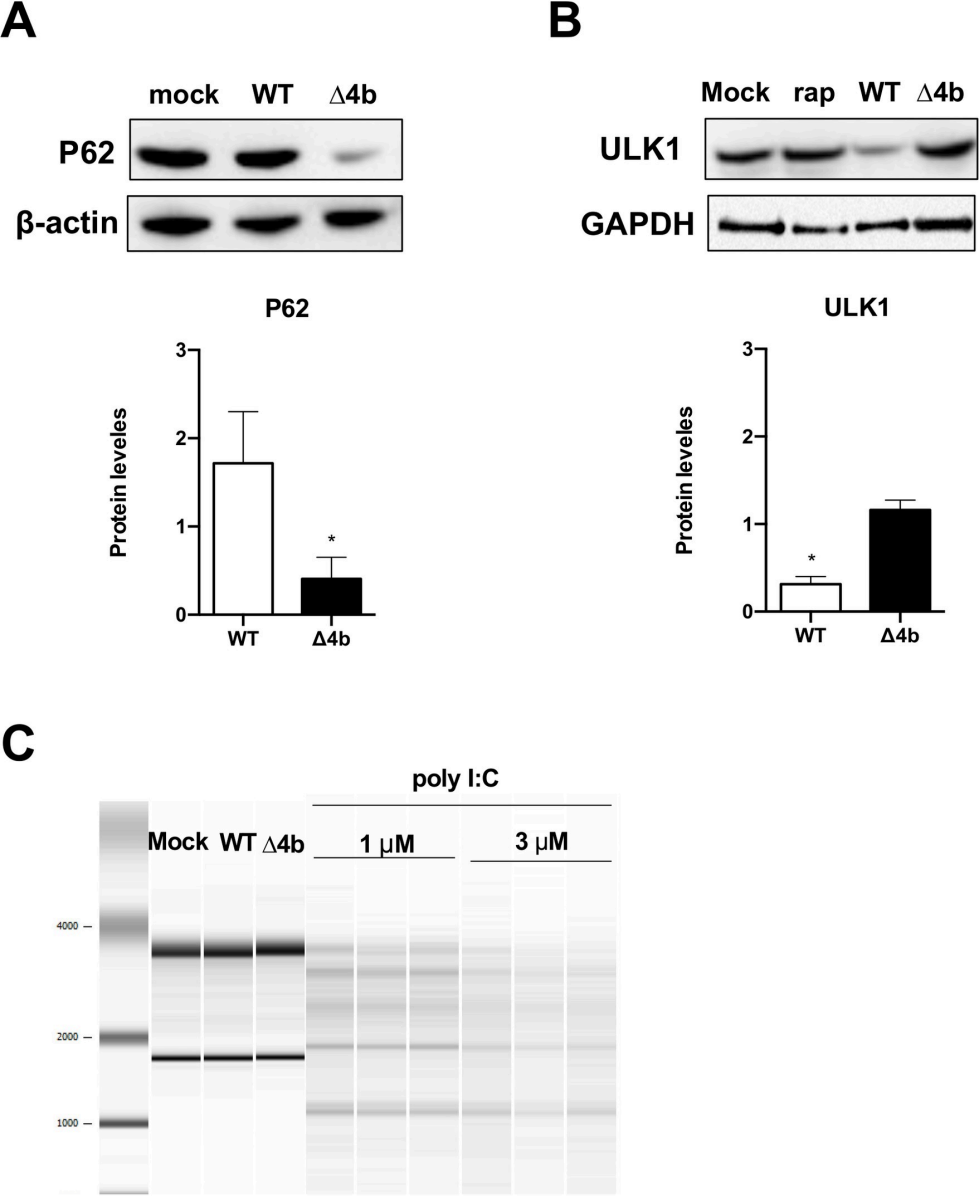
Analysis of autophagy pathway during infection with MERS-CoV-MA-WT or MERS-CoV-MA-Δ4b viruses in MRC-5 cells. MRC-5 cells were mock-infected or infected with the WT or Δ4b viruses at a m.o.i. of 1. The amount of p62 (A) and ULK1 (B) at 24 h p.i. was quantified from Western blots. Actin or GAPDH were used as loading controls. (C) Analysis of rRNA degradation pattern at 24 h p.i. or post-transfection with poly I:C in MRC-5 cells using a Bioanalyzer. Representative results from one experiment out of three (m.o.i. of 1 PFU; *n* = 3) are shown.

To study whether MERS-CoV-MA 4b protein activated or inhibited autophagy, p62 protein levels were evaluated by Western blotting in MRC-5 cells infected with MERS-CoV-MA-WT or MERS-CoV-MA-Δ4b at 24 h p.i. The reduction of p62 autophagy substrate levels is typically associated with enhanced autophagy [36] and reduced activation of NF-Kβ transcription factor [37]. A statistically significant 4.2-fold decrease in p62 protein amount was observed in cells infected with MERS-CoV-MA-Δ4b as compared to MERS-CoV-MA-WT **(Fig 5A).** Additionally, protein levels of the autophagy inductor ULK1 were increased after infection with MERS-CoV-MA-Δ4b as compared to MERS-CoV-MA-WT **(Fig 5B)**. Altogether, these data were consistent with *in vivo* observations **(Fig 3B and 3C)** and confirmed the contribution of 4b protein to the inhibition of autophagy pathway via UKL1. Concomitantly to autophagy activation in MERS-CoV-MA-Δ4b infection, a significant decrease in the pro-inflammatory response was observed in MRC5 cells, including the expression of IFN-ß, NF-kB dependent cytokines (IL-6 and IL-8) and chemokines (CCL2 and CXCL10) **(Fig 4)** [38].

4b is a non-structural protein including two known functional domains, the NLS [17, 39] and the phosphodiesterase domain (PDE) [40]. MERS-CoV 4b protein showed phosphodiesterase activity in A549 cells expressing the DPP4 receptor (A549^DPP4^), leading to the inhibition of host RNase L [41], which is an IFN-induced antiviral mechanism. Since RNaseL was described to trigger autophagy in viral infections, [42, 43], the PDE domain of 4b protein was proposed as the leading candidate for autophagy regulation in MERS-CoV [29]. To evaluate this possibility, RNase L activation was evaluated in MRC-5 cells infected with MERS-CoV-MA-WT or MERS-CoV-MA-Δ4b at 24 h p.i. (m.o.i. 1) by analyzing rRNA degradation using a Bioanalyzer instrument. RNase L-specific degradation of rRNA was not observed during infection with MERS-CoV-MA-WT or MERS-CoV-MA-Δ4b. However, RNA degradation was observed in MRC5 cells transfected with different doses of the IFN-inducer poly I:C, indicating that the RNase L pathway was functional in this cell line, although it was not activated during MERS-CoV-MA infection **(Fig 5C)**. According to these results, autophagy induced by MERS-CoV-MA-Δ4b in MRC5 cells was not associated with RNase L activity **(Fig 5C).**

### Contribution of MERS-CoV-MA 4b protein to the inflammatory response in MRC-5 cells in which the autophagy flux was chemically inhibited

**Fig 6:**
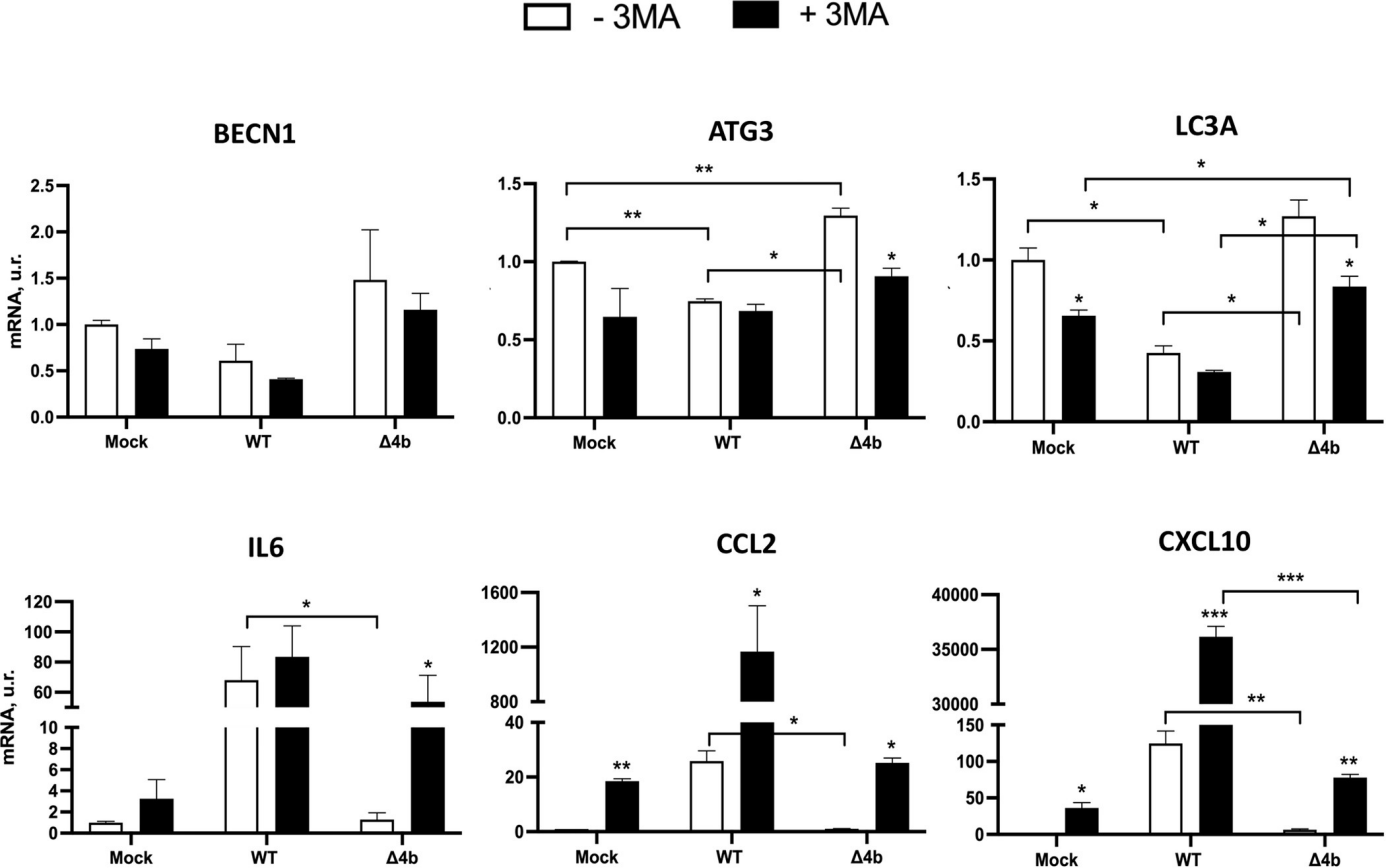
Innate immune response and autophagy flux in MERS-CoV-MA-WT and MERS-CoV-MA-Δ4b infected MRC-5 cells after the autophagy pathway was chemically inhibited. mRNA expression levels of genes related to autophagy pathway and pro-inflammatory response were quantified by RT-qPCR in MRC-5 cells either mock-infected or infected with MERS-CoV-MA-WT or MERS-CoV-MA-Δ4b at a m.o.i of 1 for 24 h p.i after the treatment (+ 3-MA) or not (- 3-MA) with 5mM 3-methyladenine for 16 h. Bars represent means and error bars represent standard deviations (n = 3). Mock, Mock-infected cells. Differences were analyzed by Student’s t test: *, p-value < 0.05; **, p-value < 0.01; ***, p-value < 0.001. Asterisks on the columns represent significant differences between mock-treated and cells.

To confirm whether MERS-CoV 4b protein was modulating the autophagy flux, the early stage inhibitor of autophagy 3-MA (5 mM) [44] was used in MRC-5 cells for 16 h before MERS-CoV infection. As expected, this treatment reduced BECN1, ATG3 and LC3A mRNA levels in mock-infected MRC-5 cells, although only LC3A decrease was statistically significant **(Fig 6)**. In non-treated cells, infection with MERS-CoV-MA-WT significantly reduced ATG3 and LC3A mRNA levels, while MERS-CoV-MA-Δ4b-infection increased ATG3 mRNA levels **(Fig 6)**. These results are in agreement with the increase in p62 accumulation and the decrease in ULK1 protein observed in MERS-CoV-MA-WT-infected MRC5 cells, as compared to MERS-CoV-MA-Δ4b infection **(Fig 5A and 5B)**, suggesting that 4b was contributing to autophagy inhibition. When autophagy in MRC5 was chemically inhibited, infection with MERS-CoV-MA-WT did not induce further changes in BECN1, ATG3 or LC3A mRNA levels, as compared to non-treated cells **(Fig 6, WT columns)**. In contrast, MERS-CoV-MA-Δ4b infection significantly increased LC3A mRNA levels, as compared to cells either mock-infected or infected with MERS-CoV-MA-WT **(Fig 6, Δ4b columns)**. Together, these results suggested that infection with MERS-CoV-MA-Δ4b was restoring the autophagy flux inhibited by 3-MA. Restoration of autophagy flux was dependent on the absence of 4b during infection, since the presence of 4b in MERS-CoV-MA-WT infected cells prevented the recovery of BECN1, ATG3 or LC3A mRNA levels. Inhibition of the autophagy flux by 3-MA increased the inflammatory response both in non-infected and MERS-CoV-MA-WT-infected MRC-5 cells. Accordingly, the partial reversion of autophagy flux induced by MERS-CoV-MA-Δ4b in 3-MA-treated cells was accompanied by an increase in the expression of pro-inflammatory cytokines (IL6, CCL2 and CXCL10) as compared to non-treated cells **(Fig 6)**.

## Discussion

The present manuscript shows that MERS-CoV-MA 4b protein is a virulence factor *in vivo* and analyzes its contribution to viral pathogenesis in hDPP4-KI mice. Deletion of 4b gene from the MERS-CoV-MA genome, using a reverse genetics system, resulted in a completely attenuated phenotype with a survival of 100% infected mice and less inflammatory pathology in their lungs. No significant differences were observed in the viral titers of MERS-CoV-MA-WT and MERS-CoV-MA-Δ4b in the lungs of mice, indicating that attenuation of MERS-CoV-MA-Δ4b was not primary caused by a reduced viral growth. Attenuation of MERS-CoV-MA-Δ4b was mainly associated with lower expression of pro-inflammatory chemokines. Additionally, higher expression levels of autophagy genes BECN1, ATG3 and LC3A were observed in the lungs of MERS-CoV-MA-Δ4b-infected mice at 4 and 6 dpi.

Deletion of the nuclear localization signal of 4b protein in MERS-CoV-MA-mNLS induced a virulent phenotype with 100% mortality in mice, similarly to WT virus. This result suggested that the pathology associated with 4b protein did not depend on its nuclear localization. The presence of 4b in the nucleus in MERS-CoV-MA-WT *in vivo* infection partially changed the pro-inflammatory cytokine profile, by upregulating IFNß, IL-6 and IL-10, as compared to MERS-CoV-MA-mNLS, which expressed a cytoplasmic 4b. However, these differences were not enough to attenuate MERS-CoV-MA-mNLS. A common cellular response observed when 4b was expressed either in the nucleus or the cytoplasm, mainly consisting in increased levels of some pro-inflammatory cytokines (CCL2, CCL4, CXCL1, CXCL2, CXCL10 and CXCL12) and autophagy inhibition, led to 100% mortality of mice infected with MERS-CoV-MA-WT or MERS-CoV-MA-mNLS.

In the hDPP4-KI mouse model, infection with MERS-CoV-MA-WT moderately increased IFN-β levels at 4 and 6 dpi in contrast to almost undetectable levels observed in the absence of protein 4b or in the presence of cytoplasmic 4b-mNLS, suggesting that 4b nuclear location was required to induce IFN-ß production. Other authors proposed different functions for 4b depending on its subcellular location. Cytoplasmic location was related to the regulation of IFN-β production [45] and antagonism of the OAS-RNase L pathway [46]. Our results cannot exclude that in mice infected with MERS-CoV-MA-Δ4b, IFN-ß expression was increased earlier in infection (before 4 dpi) and decreased later, at 4 and 6 dpi. Late or prolonged IFN-β response was described to decrease survival in MERS-CoV infected mice, while early IFN-ß production was associated with protection [32]. An early increase in IFN-β levels in MERS-CoV-MA-Δ4b could explain the lower levels of chemokines CXCL10 and CCL2 in lungs at 4 dpi, as previously proposed [32]. Other authors have described an IFNß antagonism function for protein 4b when overexpressed in the absence of infection [7] in contrast to the reduced IFNß levels observed after infection with MERS-CoV-MA-Δ4b in mice (Fig 3) or MRC-5 cells (Fig 4B). These different results might be explained by the overexpression of a single protein at non-physiological levels in the absence of other viral proteins. This ectopic expression does not acknowledge the complex interplay of proteins during virus replication. Another paper showed that, in A549 cells at 24 and 36 h p.i., deletion of 4b gene from MERS-CoV did not change IFNß expression as compared to MERS-CoV-WT, while significantly increased in IFNλ [41]. Differences in cell lines and in the times post infection selected in the different studies might justify the diversity observed in the contribution of 4b protein to the innate immune response in mice and cell cultures [5, 17].

Infection with the attenuated MERS-CoV-Δ3–5 reduced the expression of pro-inflammatory cytokines *in vivo* [5], although this phenotype could not be associated with any specific accessory protein. MERS-CoV pathology is associated with abundant cellular infiltrates in the lungs of mice [47, 48]. Therefore, it is not surprising that reduced levels of chemokines were detected in the lungs of mice infected with the attenuated MERS-CoV-MA-Δ4b, in contrast to the elevated levels of CCL2, CCL4, CXCL1, CXCL2 and CXCL10 observed in the lungs infected with virulent MERS-CoV-MA-WT and MERS-CoV-MA-mNLS viruses.

Autophagy is a host mechanism regulated by some viruses, including CoVs, during infection. However, it is still unclear how CoVs regulate the autophagy machinery [28, 49]. Mouse hepatitis virus (MHV) induces autophagy for the formation of replication complexes [50], while SARS-CoV induces an incomplete autophagy process by accumulating autophagosomes and blocking the fusion of autophagosomes with lysosomes [51]. One study showed that 4b and 5 MERS-CoV accessory proteins limit autophagy when expressed ectopically by blocking the fusion of the autophagosome with the lysosome. Moreover, deletion of 4b or 5 genes from the viral genome led to autophagy enhancement, suggesting that both proteins contribute to the inhibition of the autophagic flux during infection. Reduced viral growth in these MERS-CoV deletion mutants of 4b or 5 suggested that autophagy inhibition might benefit viral growth [29]. In agreement with this idea, another paper described that kinase inhibitors of PI3K/AKT/mTOR pathway, which might promote autophagy, inhibited MERS-CoV growth in Huh-7 cells [52]. This study provides indirect evidence of increased autophagy in the absence of 4b protein in MRC-5 infected cells, as shown by the decrease in the levels of p62 substrate and the increase in ULK1 protein when 4b protein was deleted from the viral genome. Accordingly, *in vivo* infection with MERS-CoV-MA-Δ4b increased the expression of autophagy mediators BECN1, ATG3 and LC3A. In addition, the autophagy inhibition observed during MRC-5 infection was not a consequence of the 4b phosphodiesterase described [41] in human airway epithelium-derived A549 cells.

In addition to the potential effect of autophagy in viral growth, a protective role by reducing the excessive secretion of cytokines [53, 54] has been described. Since the severity of CoV-induced lung disease is associated with exacerbated inflammation [55–58], the activation of autophagy through the inhibition of mTOR would protect against MERS-CoV pathogenesis, as shown in this manuscript using MERS-CoV-MA-Δ4b.

**Fig 7:**
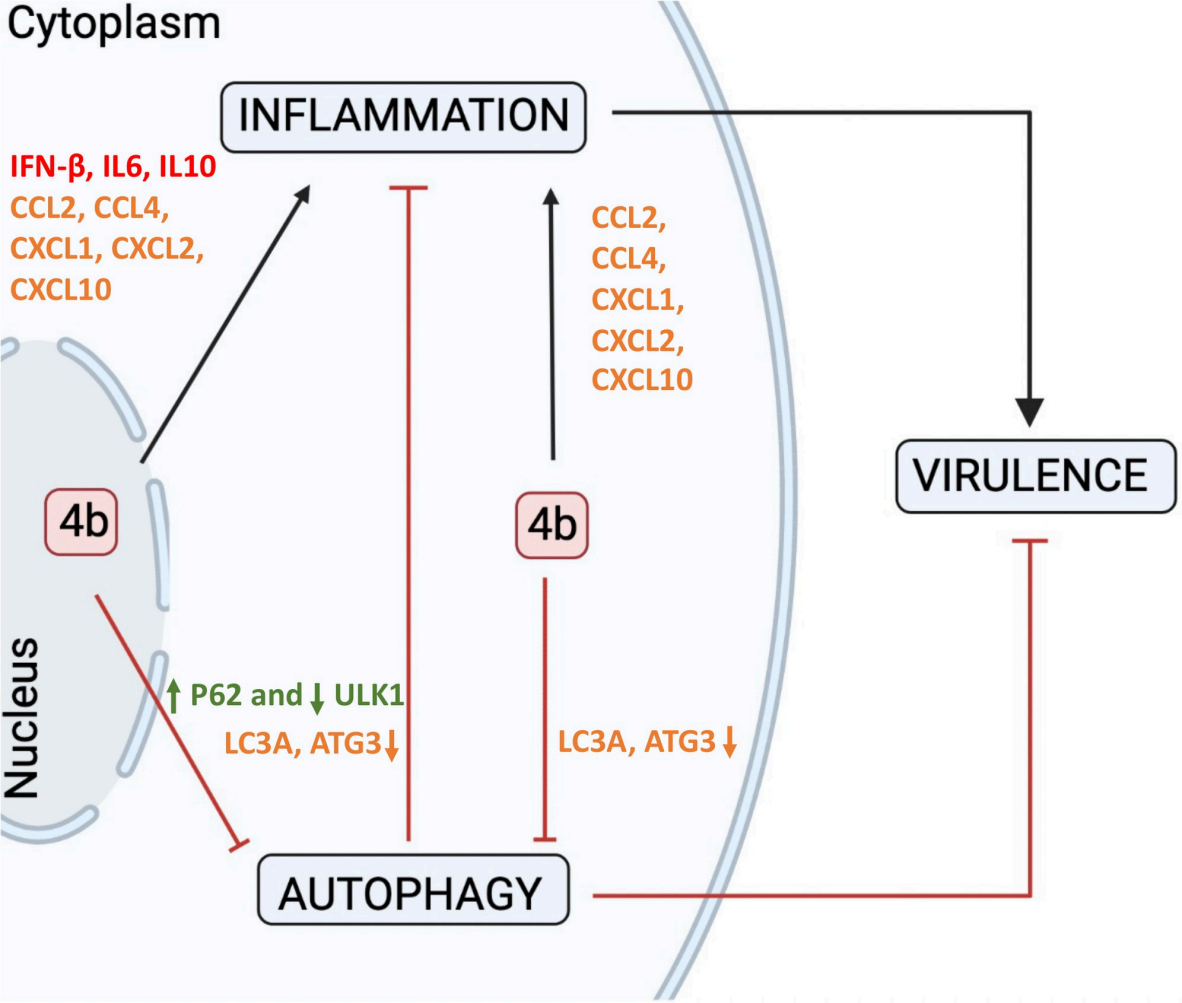
Proposed mechanism of action of 4b protein in MERS-CoV virulence. During MERS-CoV-WT infection, 4b protein accumulates in the nucleus, while in the absence of a functional nuclear localization signal (mNLS), 4b protein is retained in the cytoplasm. The presence of either nuclear or cytoplasmatic 4b is associated with activation of inflammation and inhibition of autophagy, which contribute to virulence in vivo, as shown for MERS-CoV- WT and MERS-CoV-mNLS in a mouse model of infection. According to this work, autophagy contributes to limit inflammation. In the absence of 4b protein, autophagy was activated and a lower inflammatory response was induced, which contributes to attenuation. Chemokine mRNAs increased both in MERS-CoV-WT and MERS-CoV-mNLS infection are indicated in orange. Cytokines mRNA differentially induced in MERS-CoV-WT infection are shown in red. Changes in protein levels are indicated in green.

Considering the results presented in this manuscript, attenuation of MERS-CoV-Δ4b was accompanied by a decrease in the inflammatory response and an activation of autophagy. Therefore, we are tempted to propose that 4b protein is a virulence factor in MERS-CoV infection acting by inducing inflammation and inhibiting autophagy. Repression of autophagy might contribute to virulence either by promoting viral growth [29] or by increasing inflammation **(Fig 7)**. Therefore, the autophagy pathway might represent a potential target for host-directed antivirals and drugs that promote autophagy and might be useful to design anti-coronavirus therapies.

## Material and methods

### Ethics statement

All work with MERS-CoV-MA infected animals was performed in the BSL3+ laboratory of the Animal Health Research Center (CISA-INIA/CSIC; Valdeolmos, Madrid, Spain). Infected mice were housed in a self-contained ventilated rack (Allentown, NJ). Experimental protocols were approved by the Environmental Council of Madrid (permit number PROEX 112/14) and the Ethical Committee of the Animal Health Research Center (CISA-INIA/CSIC) (permit numbers CBS 2014/005 and CEEA 2014/004), in strict accordance with Spanish National Royal Decree 53/2013 and the international EU Guideline 2010/63/UE about the protection of animals used for experimentation and other scientific purposes and Spanish National Law 32/2007 about animal welfare.

### Cells

Human lung fibroblast (MRC-5), baby hamster kidney (BHK-21), monkey Vero cells (CCL-81) and human liver-derived (Huh-7) cells were grown in Dulbecco’s modified Eagle medium (DMEM) supplemented with 10% fetal bovine serum (FBS), 2% glutamine and 1% non-essential amino acids (Sigma-Aldrich). All these cells were incubated at 37°C with 5% CO_2_. BHK-21 cells were used for the transfection of bacterial artificial chromosomes (BACs) including the infectious cDNA of recombinant viruses. The Huh-7 cell line was used for characterization of recombinant viruses and the MRC-5, for studying the autophagy regulation by 4b protein.

### Plasmid construction

An infectious cDNA clone encoding a mouse adapted MERS-CoV (MERS-CoV-MA) assembled in a BAC [14] was used as the background to introduce mutations in 4b gene. Recombinant viruses MERS-CoV-MA-Δ4b and mNLS were generated as previously described [17]. Briefly, genomic fragments containing the deletion of 4b (Δ4b) and the mutation of the nuclear localization signal (mNLS) were inserted at PacI and NheI restriction sites, corresponding to positions 25851 and 26945, respectively, in the MERS-CoV-MA genome. The genetic integrity of MERS-CoV-MA infectious cDNA was verified by restriction analysis and Sanger sequencing.

### Transfection and recovery of infectious MERS-CoV-MA viruses from cDNA clones

All recombinant viruses were rescued from infectious cDNA clones as previously described [6]. Briefly, BHK cells grown in 12.5 cm^2^ flasks were transfected with 6 μg of each pBAC using Lipofectamine 2000 (Invitrogen). At 6 h post-transfection, BHK cells were trypsinized and plated over confluent Huh-7 cells. Supernatants containing infectious viruses were collected (passage 0) after 3 days and passaged on fresh Huh-7 cells (passage 1).

### Virus titration

Virus titers were determined by plaque assay on VERO-81 cells grown to 90% confluence [17]. Briefly, after 1 h of adsorption with serial dilutions of viruses, cells were overlaid with DMEM containing 0.6% low-melting agarose and 2% FBS. After 72 h incubation at 37°C, cells were fixed with 10% formaldehyde and stained with 0.1% crystal violet. All the infections with the recombinant viruses generated in this work were performed in biosafety level 3 facilities at CNB-CSIC according to the biosafety guidelines and standard procedures.

### Growth kinetics

Confluent Huh-7 and MRC-5 cells were infected at a m.o.i. of 0.1 and 0.001 PFU/cell and cell supernatants were collected at 24, 48, 72 and 96 h p.i. Virus titers were determined by plaque assay as described above.

### Mice infection and virus virulence *in vivo*

MERS-CoV–susceptible knock-in C57BL/6 mice genetically modified to express the MERS-CoV receptor (hDPP4-KI mice) [59] were provided by Dr Paul McCray (University of Iowa, Iowa, USA) and a breeding colony was established at the CNB-CSIC animal facility (Madrid, Spain). A total of 44 hDPP4-KI 30-week-old female mice (11 mice per group) were anesthetized with isoflurane and intranasally inoculated with 5x10^4^ PFU/animal of each virus in 50 μl of DMEM. Mock-infected mice were intranasally inoculated with 50 μl DMEM. Weight loss and mortality were monitored daily for 12 days in those mice whose euthanasia had not been scheduled at 3 and 6 dpi. Mice with a body-weight reduction of 20% or more were euthanized to meet the endpoint criteria. At 4 and 6 dpi, n = 3 mice per group were sacrificed for lung collection. To analyze viral titers, one half of the right lung (including the three lobes) was homogenized in Phosphate Buffered Saline (PBS) containing 100 IU/ml penicillin, 100 μg/ml streptomycin, 50 μg/ml gentamicin and 0.5 μg/ml fungizone using the MACS homogenizer (MiltentyBiotec) according to the manufacturer’s protocols. The second half of right lung was homogenized in RLT Buffer with 1% of β-mercaptoethanol for RNA extraction.

### Lung histopathology

To examine lung histopathology, left lungs of mice were immersion fixed in 10% zinc formalin for 24 hours. After fixation period, samples were routinely processed and embedded in paraffin blocks. Serial 5-μm sections were stained with hematoxylin and eosin (H&E). The histopathological evaluation of lung sections was carried out using an Olympus BX43 microscope by a single veterinary pathologist who was blinded to the identity of mice (Veterinary Pathology Department, Animal Health Research Center-CISA, Valdeolmos, Madrid, Spain). To assess the presence and severity of histopathological lesions, lung inflammation parameters based on previous reports on SARS-CoV-2 infection in mouse models were used [60]. The histopathological parameters evaluated were: alveolar haemorrhages, alveolar edema, perivascular edema; alveolar septal thickening (interstitial pneumonia), inflammatory cell infiltration in alveoli, bronchi/bronchioles with epithelial necrosis, detached epithelium or inflammatory cells in the lumen (bronchitis/bronchiolitis); peribronchial/peribronchiolar and perivascular mononuclear infiltrates, cytopathic effect in pneumocytes or syncytia. The histopathological parameters were graded following a semi-quantitative scoring system as follows: (0) no lesion; (1) minimal lesion; (2) mild lesion; (3) moderate lesion; (4) severe lesion. The cumulative scores of histopathological lesions provided the total score per animal. Populations of lung-infiltrated inflammatory monocyte-macrophages or lymphocytes were identified according to their morphological differences (size and shape of the whole cell and the nucleus) and additional histological characteristics.

### Gene expression analysis by RT-qPCR

Total RNA was isolated from culture cells or homogenized lungs using RNeasy mini kit (Qiagen) following manufacturer’s specifications. Total cDNA was synthesized using 100 ng of total RNA as a template, random hexamers, and the High-Capacity cDNA reverse transcription kit (Invitrogen). To quantify mRNA levels, commercially available Taqman assays were used **(Table 1)**. Human hydroxymethylbilane synthase (HMBS) gene (Hs00609297_m1) or rRNA 18S (Mm03928990_g1) were used as endogenous controls for normalization in human cell cultures or mouse tissues, respectively. Data were acquired with an ABI Prism 7500 sequence detection system (Applied Biosystems) and analyzed with ABI PRISM 7500 SDS version 1.2.3 software. Relative quantifications were performed using the 2^-ΔΔCt^ method [61].

**Table 1:** Taqman assays.

Species	Gen symbol	Description	Assay ID
**Mouse**	ATG3	Autophagy related 3	Mm00471287_m1
	BCN1	Beclin 1	Mm01265461_m1
	CCL2	Monocyte chemotactic protein 1 (MCP-1)	Mm00441242_m1
	CCL4	Macrophage inflammatory protein 1β (MIP-1β)	Mm00443111_m1
	CCL5	C-C motif chemokine 5, RANTES	Mm01302428_m1
	CXCL1	C-X-C motif chemokine ligand 1	Mm04207460_m1
	CXCL2	C-X-C motif chemokine ligand 2	Mm00436450_m1
	CXCL10	Interferon gamma-induced protein 10 (IP-10)	Mm00445235-m1
	CXCL12	C-X-C Motif Chemokine Ligand 12	Mm00445553_m1
	IFN-β	Interferon beta (IFN-β)	Mm00439552_s1
		IFN- γ	Interferon gamma (IFN-γ)	Mm01168134-m1
	IL-6	Interleukin-6	Mm00446190_m1
	IL-10	Interleukin-10	Mm00439614_m1
	ISG15	Ubiquitin-like protein ISG15	Mm01705338_s1
	LC3A	Microtubule-associated protein 1 light chain 3 alpha	Mm00458724_m1
**Human**	CCL2	Monocyte chemotactic protein 1 (MCP-1)	Hs00234140_m1
	CXCL10	Interferon gamma induced protein 10 (IP-10)	Hs00171042_m1
	IFN-β	Interferon beta (IFN-β)	Hs02621180_s1
	IL-6	Interleukin-6	Hs00985641_m1
	IL-8	Interleukin-8	Hs99999034_m1
	TNF-α	Tumor necrosis factor alpha (TNF-α)	Hs99999043_m1

### Immunoblotting

Cell extracts were lysed in sample buffer containing dithiothreitol (DTT) and β-mercaptoethanol (Life Technologies). Boiled samples were separated by SDS-polyacrilamyde gel electrophoresis (PAGE) in 4–12% Bis/Tris Precast Gels (LifeTechnologies) and then transferred to polyvinylidene difluoride (PVDF) membranes. Membranes were blocked with 3% milk and probed with primary antibodies: anti-p62 rabbit polyclonal antibody (Cell SignalingTechnology#5114) (1:1000), anti-ULK1 rabbit monoclonal antibody (Sigma-Aldrich#A7481) (1:1000) and anti-β-actin rabbit monoclonal antibody (1:5000) (Cell Signaling Technology #8457). Anti-rabbit secondary antibodies labelled with horseradish peroxidase (HRP) (Sigma-Aldrich #A0545)(1:10000) were used for detection. Proteins were visualized using a chemiluminescent substrate for HRP (Clarity Western ECL Substrate, Bio-Rad) and the ChemiDoc XRS+ System (Bio-Rad).

### Indirect immunofluorescence assay

Huh-7 cells grown on sterile glass coverslips were infected at a m.o.i. of 0.1 with each virus. At 24 h p.i., cells were fixed with 4% paraformaldehyde in PBS for 45 min and permeabilized with methanol for 10 min at 20° C. Then, cells were blocked with 10% FBS in PBS at 20° C for 1h. 4b protein was stained with a specific polyclonal antiserum generated in rabbit, as previously described [17]. After 1h incubation, coverslips were washed with PBS and incubated at RT for 45 min with Alexa Fluor 488 conjugated secondary antibodies (Invitrogen) diluted 1:500 in PBS-5% FBS. Finally, coverslips were mounted in ProLong Gold antifade reagent (Invitrogen) and imaged on a Leica SP8 confocal microscope.

### Analyses of RNase L-mediated rRNA degradation

Total RNA from MRC-5 cells either mock-infected or infected with MERS-CoV-MA-WT or MERS-CoV-MA-Δ4b at m.o.i. 1 was harvested with the RNeasy kit (Qiagen, Valencia, CA) 24 h p.i.. RNA integrity was analyzed on the Agilent 2100 Bioanalyzer system. As a positive control for the activation of RNaseL pathway in MRC-5 cell line, the RNA from MRC-5 cells transfected with Poly I:C (1 or 3 μM) was harvested and analyzed using the same protocol.

### Chemical inhibition of autophagy pathway

To determine the effect of inhibiting the autophagic flux on the MERS-CoV-induced inflammatory response, the early stage inhibitor of autophagy 3-methylalanine (3-MA) was used, following the guidelines for monitoring autophagy [44]. MRC-5 cells were untreated or treated with 5 mM 3-MA for 16 h. After the treatment, supernantants were removed, the cells were washed three-times with PBS and either mock-infected or infected with MERS-CoV-MA-WT or MERS-CoV-MA-Δ4b at m.o.i. 1 for 24 h. Total RNA from MRC-5 cells was harvested and mRNA expression levels of autophagy genes (BECN1, ATG3 and LC3A) and pro-inflammatory cytokines (IL6, CCL2 and CXCL10) were evaluated by qRT-PCR.

### Statistical analysis

Two-tailed, unpaired Student’s *t* tests were used to analyze differences in mean values between groups. All results were expressed as means ± standard deviations. Differences between groups were considered statistically significant in cell lines experiments when P value was less than 0.05 (p<0.05, *; p<0.01, **) or less than 0.1 in *in vivo* experiments (p<0.1, *; p<0.01, **) [62].
